# The efficacy of computer-assisted cognitive behavioral therapy (cCBT) on psychobiological responses and perioperative outcomes in patients undergoing functional endoscopic sinus surgery: a randomized controlled trial

**DOI:** 10.1186/s13741-021-00195-3

**Published:** 2021-08-19

**Authors:** Yang Yang, Yuling Li, Haibin Zhang, Yong Xu, Binquan Wang

**Affiliations:** 1 Nursing College, Shanxi Medical University, Taiyuan, People’s Republic of China; 2 Nursing Department, The First Hospital of Shanxi Medical University, Taiyuan, People’s Republic of China; 3 Department of Anesthesia, The First Hospital of Shanxi Medical University, Taiyuan, People’s Republic of China; 4 Department of Psychiatry, The First Hospital of Shanxi Medical University, Taiyuan, People’s Republic of China; 5 Shanxi Key Laboratory of Artificial Intelligence Assisted Diagnosis and Treatment for Mental Disorder, The First Hospital of Shanxi Medical University, Taiyuan, People’s Republic of China; 6 Department of Otorhinolaryngology Head and Neck Surgery, The First Hospital of Shanxi Medical University, Taiyuan, People’s Republic of China; 7 Shanxi Key Laboratory of Otorhinolaryngology Head and Neck Cancer, The First Hospital of Shanxi Medical University, Taiyuan, Shanxi People’s Republic of China

**Keywords:** Computer-assisted cognitive behavioral therapy, Psychobiological response, Functional endoscopic sinus surgery, Anxiety, Insomnia, Cortisol

## Abstract

**Background:**

Functional endoscopic sinus surgery (FESS) is required to minimize bleeding to maintain a clear operative field during surgery, so it is important to preoperative anti-anxiety and stable hemodynamics. Initial evidence suggests cognitive behavioral therapy (CBT) is effective to minimize surgery-related stress and to speed up recovery. The study aimed to evaluate the efficacy of a newly developed computer-assisted CBT (cCBT) program on surgery-related psychobiological responses in patients undergoing FESS.

**Methods:**

Participants were allocated to a CCBT group (cCBT; *n* = 50) or a UC group (usual care; *n* = 50) by random number table. The State Anxiety Inventory (SAI), Patients Health Questionnaire-9 (PHQ-9), Athens Insomnia Scale (AIS), systolic blood pressure (SBP), diastolic blood pressure (DBP), and heart rate (HR) were assessed before intervention (T1), at 1 h before operation (T2), at postoperative 48 h (T3), and 96 h (T4: after intervention completed) respectively. The stress hormone was assayed at T1 and T2. The duration of surgery, anesthesia, and post-anesthesia care unit (PACU) were recorded. A satisfaction survey about nursing services was completed by participants before discharge.

**Results:**

Compared to the UC group, the SAI scores at T2 and the AIS scores at T3 were lower in the CCBT group (*p <* 0.01 and *p* = 0.002). The positive rate of participants who were moderate and severe anxiety (SAI score > 37) at T2 were lower (72% vs. 88%, *p* = 0.04); the cortisol levels, SBP, DBP, and HR at T2 in the CCBT group were lower (*p* = 0.019 and all *p* < 0.01); the duration of anesthesia and PACU was shorter (*p* = 0.001 and *p* < 0.01); the CCBT group showed higher satisfaction scores.

**Conclusion:**

The newly developed cCBT program was an effective non-pharmacological adjunctive treatment for improving the surgery-related psychosomatic responses and perioperative outcomes.

**Trial registration:**

The study was registered with the Chinese Clinical Trial Registry (ChiCTR1900025994) on 17 September 2019.

**Supplementary Information:**

The online version contains supplementary material available at 10.1186/s13741-021-00195-3.

## Background

Chronic rhinosinusitis (CRS) is a chronic local inflammatory disease with a relatively high incidence. The overall prevalence of CRS was 8.0% in seven cities in mainland China (Shi et al. 2015). Once CRS becomes refractory and no longer responsive to medication, functional endoscopic sinus surgery (FESS) represents the first choice for surgical treatment (Kaper et al. 2020). Minimizing blood loss and achieving the optimal intraoperative surgical field visibility are two important determinants of surgical success and outcome in FESS (Chhabra et al. 2020). Perioperative hemodynamic management can optimize surgical conditions, minimize bleeding, and optimize patient outcomes in FESS (Martin et al. 2020). Preoperative anxiety influences physiological outputs including increased heart rate, decreased heart rate variability, blunted post-stress systolic blood pressure recovery, and elevated levels of cortisol (Epel, 2020, Landon et al. 2019).

The studies on controlled hypotension and reducing intraoperative bleeding are mainly focused on the comparison of total intravenous anesthesia (TIVA) and inhalational anesthesia (IA) (Lu et al. 2020) and the use of drugs (Chhabra et al. 2020). To minimize the adverse reaction, using properly non-invasive and non-pharmaceutical healing techniques may be an additional approach. And the perioperative psychological interventions based on multidisciplinary therapy have proved to be an effective and adequate strategy (Visioni et al. 2018). The cognitive-behavioral therapy in psychological interventions was proved to influence positively on modulating the surgical stress response and improving surgical outcomes, particularly in those with maladaptive features (Villa et al. 2020).

Cognitive-behavioral therapy (CBT) is the first choice recommended by the FDA for the non-pharmacologic treatment of depression and anxiety disorders, which focuses on correcting maladaptive behavior and negative thoughts (Beck, 1964). CBT had been proved feasible and acceptable to improve symptoms of depression or anxiety, health-related quality of life (HRQOL) in patients undergoing surgery (Dao et al. 2011). Multiple barriers prevent patients undergoing major surgery from accessing the CBT, such as a shortage of trained therapists, high costs, and a lack of accessibility in remote areas, which contribute to lacking patient engagement (Cartreine et al. 2010). The computer-assisted cognitive behavioral therapy (cCBT) had been developed as effective psychotherapy and reveals promise for overcoming barriers in medical settings. cCBT has been suggested as an attractive alternative treatment to face-to-face treatment, which is a remote mental-health intervention model and presents structured sessions of CBT via a computer interface or smartphone application (Carlbring et al. 2018). The overall results of RCT suggest that cCBT can be an effective treatment for depression and health anxiety in primary care (Andrews et al. 2018). Moreover, it can be a useful adjunct treatment for somatic conditions with anxiety and depression (Wright et al. 2018). However, the implementation of cCBT in ameliorating negative emotions and somatic distress associated with surgery is limited. There are few study reports on cCBT intervention in patients with FESS.

Accordingly, the current study aimed to conduct an RCT to test the efficacy of a newly developed cCBT program on improving psychological and physical parameters compared to a matched control condition. The hypotheses of the study are as follows: the patients of the CCBT group will express more satisfying subjective perception and show objective indications in favor of treatment and rehabilitation than the control group, such as less preoperative anxiety, postoperative depression, insomnia, cortisol, and more stable hemodynamics (BP and HR).

## Methods

### Study design

The present study was a prospective, randomized, controlled, and single-blind trial with one experimental arm (CCBT group) and one control arm (UC group). Participants were allocated one of two groups according to random number table: either five sessions of cCBT or health education based on usual care. Participants were recruited from the Otolaryngology Department through the First Hospital of Shanxi Medical University from 20 September 2019 to 10 January 2020. The patient, attending anesthesiologist and surgeon were allocated blindly to the study groups. Surgeons were made up of two teams which were allocated task randomly. Anesthesiologist is a specialized doctor whose clinical working time is more than 10 years and responsible for the anesthesia of all participants.

### Anesthesia technique

The anesthesia way was TIVA with routine paralysis and endotracheal intubation. Anesthesia was induced with midazolam (0.03 mg/kg), etomidate (0.3 mg/kg), sufentanil (0.5 μg/kg), and cisatracurium (0.15 mg/kg) to facilitate oral intubation. The anesthesia was maintained with a continuous infusion of propofol infusion (4 ~ 6 mg/kg/h) and remifentanil (0.1 ~ 0.3 μg/kg/min) by a target-controlled infusion (TCI) to maintain bispectral index (BIS) at 40 to 60. A controlled hypotension was provided and adjusted according inter-operative bleeding (target: SBP up to 80 ~ 90 mmHg, arterial pressure up to 50 ~ 70 mmHg, or a decrease up to 30% than mean arterial pressure) (Modir et al. 2018). The patient was transferred from the PACU to the general ward, if Steward score was assessed > = 4 (or the modified Aldrete score was > = 9) (Sun et al. 2017).

### Surgical technique

After induction of general anesthesia, local anesthesia and epinephrine 1:1000 soaked cotton wool pledgets were applied the nasal cavity for 5 to 10 min to reduce bleeding. The surgical technique consisted of bilateral anterior and posterior ethmoidectomy with bilateral middle meatotomy according to Messerklinger technique developed by Professor Walter Messerklinger, which only the Ostiomeatal Complex (OMC) lesions were removed without damage to the mucosa in the sinuses(Kane, 2020).

### Participants

Inclusion criteria were the following: (1) aged 18 ~ 60 years; (2) scheduled to undergo FESS within a week; (3) general anesthesia can be acceptable as a form of anesthesia; (4) American Society of Anesthesiologists (ASA) grades I and II; (5) be willing to answer the questionnaires and participate in the interventions. Exclusion criteria included the following: (1) diagnosis of definite history of psychiatric illness, substance abuse/dependence within 12 months before enrollment; (2) undergoing a surgery that was postponed for more than a week or changed to emergent surgery; (3) currently receiving any psychiatric or psychological treatment, including psychiatric medication; (4) the Patient Health Questionnaire Depression Scale-9 (PHQ-9) score ≥ 20 or the risk of suicide (5) those who had serious cardiovascular and respiratory diseases.

### Measures and outcomes

Participants were identified and confirmed from surgical-plan lists by the Electronic Medical Record System. Then, they were approached by researchers and written informed consent would be obtained according to the ethical requirements before any study procedure. The participants who met the inclusion criteria were enrolled and assessed to collect demographic information and baseline data. Two study staff were blinded to collect and analyze data separately.

Baseline characteristics included sex, age, education, principal diagnosis, surgery type, and anesthesia grading. The baseline measures (anxiety, depression, insomnia, stress hormones, vital signs) were collected on the day after hospitalization (before intervention: T1). The state anxiety, stress hormones, and vital signs were re-obtained from patients 1 h before surgery (T2). The depression, insomnia, and vital signs were re-assessed at postoperative 48 h (T3). After all interventions are completed, anxiety, depression, insomnia, and vital signs were re-assessed at postoperative 96 h (T4). And a satisfaction survey about nursing services was completed by participants before discharge. The duration of surgery, anesthesia, and post-anesthesia care unit (PACU) were recorded.

State-Trait Anxiety Inventory (STAI) was developed by Spielberger C.D and Gorsuch R.L (Spielberger et al. 1983). State Anxiety Inventory (SAI) assesses current state of anxiety at a particular moment and Trait Anxiety Inventory (TAI) evaluates relatively stable aspects of “anxiety proneness” (Spielberger et al. 1983). These scale were reliable, and Cronbach’s alpha = 0.80 and 0.85 for SAI and TAI for the current sample. Past studies displayed that SAI score > 37 was considered to be moderate and severe anxiety (Pan et al., 2018).

The Patient Health Questionnaire Depression Scale-9 (PHQ-9) can establish grade depressive symptom severity (Kroenke et al. 2001). We obtained a value of Cronbach’s α was 0.80 for the current sample. Athens Insomnia Scale (AIS) was developed by Soldatos to quantify sleep difficulty based on ICD-10 criteria (Soldatos et al. 2000). Cronbach’s α was 0.90 for the current sample. The PHQ-9 score > 4 and the AIS score > 5 were considered a depression or insomnia clinical cutoff state (Kroenke et al. 2001, Soldatos et al. 2003).

We collected venous blood of a part of participants (60/100) between 6:00 a.m. and 8:00 a.m. to assay serum cortisol and Adrenocorticotropic Hormone (ACTH) at T1 and T2 respectively. A single venipuncture collected 5 ~ 8 ml blood into purple and yellow plain tubes (with coagulants and separation gel). The cortisol and ACTH levels were measured by chemiluminescence assay (CLIA) and microparticles CLIA.

### Study interventions

#### Introduction

The potential participants were given a brief introduction about the study program before beginning the five-session intervention, especially the importance of mental health for postoperative recovery. Eligible in-patients who were willing to participate were registered and kept in touch by study staff.

#### CCBT program intervention

The new cCBT program named “computer-assisted psychosomatic cognitive behavioral therapy during perioperative period (CPCBT-Period)” was developed and optimized based on Enhanced Recovery After Surgery (ERAS), nursing education and CBT theory. The CCBT group received the optimized cCBT intervention in addition to usual care. The cCBT is a non-internet-based computer program that includes registration and therapy conducted in a medical setting. The program is based on condition-surgery and CBT elements, which comprised five sessions, and each session took about 20 min to complete. The preoperative preparation time is usually only 3 (± 1) days for patients with FESS since admission and postoperative recovery time is about 7 (± 2) days. The time points of intervention are 2 days and 1 day before surgery and 2 days, 3 days, and 4 days after surgery. All sessions would finish before discharge. Participants in the CCBT group entered their information into the program and the first treatment was administered soon after registration. Each session starts by logging into the admission number. Next time logging in, individualized treatment will continue. The treatment modules within the program included cognitive therapy, cognitive consolidation, and behavioral relaxation therapy. The contents of perioperative education were reviewed and passed by clinical nursing specialists which were unique and customized for surgical patients. The primary components of the cognitive therapy module included the following:

Session 1: Preoperative psychological preparation;

Session 2: Preoperative physical preparation and introduction to the surgical environment;

Session 3: Management of postoperative pain, insomnia, and anti-thrombus;

Session 4: Postoperative exercise and diet;

Session 5: Education on steps following hospital discharge.

The behavioral therapy module included relaxing training, such as imaginative relaxing exercise, progressive muscle relaxation, breathing exercises, relaxing sleep exercise, and a mindfulness meditation body scan. Homework as cognitive consolidation was then provided for participants to answer questions in the form of a game, in which the questions served as a review of the previous cognitive therapy module. The cognitive therapy module and behavioral therapy module were presented via video. The contents and design were reviewed and passed by psychotherapists.

### Usual care intervention

In the UC group, patients were administered the FESS routine care conducted according to the Perioperative Care Manual and Consensus on ERAS (Chen Bing, 2018). To match the CCBT group, participants randomized to this condition, including five sessions, focused on education about the illness, surgery, anesthesia, and postoperative nursing. These sessions were developed in consultation with clinical nursing specialists. A 20-min verbal briefing per session was administered to participants by study staff. Topics included the introduction of illness and surgery; preoperative psychological and physical preparation; postoperative disease care; activities and diet after surgery; and education on steps following hospital discharge.

### Data analysis

To have a difference greater than 5 in average levels of state anxiety between groups used PASS11.0, we estimated a sample size of 46 patients per arm according to the previous value of the standard deviation σ = 10 (Ruffinengo et al. 2009). Allowing for a 10% dropout rate, approximately 50 cases were ultimately included per group.

Analyses of the outcomes were conducted by IBM SPSS Statistics, version 23.0 (2010 SPSS Inc., IBM Company, Armonk, NY, USA). Descriptive statistics (percentages, means, and standard deviations [SD]) were used to summarize baseline participant characteristics and scores on the self-report measures. Chi-square and Student’s *t* tests were performed to test differences of between-group. The repeated-measures ANOVA models were conducted to analyze the changes from baseline in the outcome measures. Median and interquartile intervals were used to describe data of non-normal distribution (Satisfaction) and the Wilcoxon rank-sum test was used for comparisons between groups. All of the tests were two-sided, and statistical significance was set at *p* < 0.05.

## Results

### Sample characteristics

**Fig. 1:**
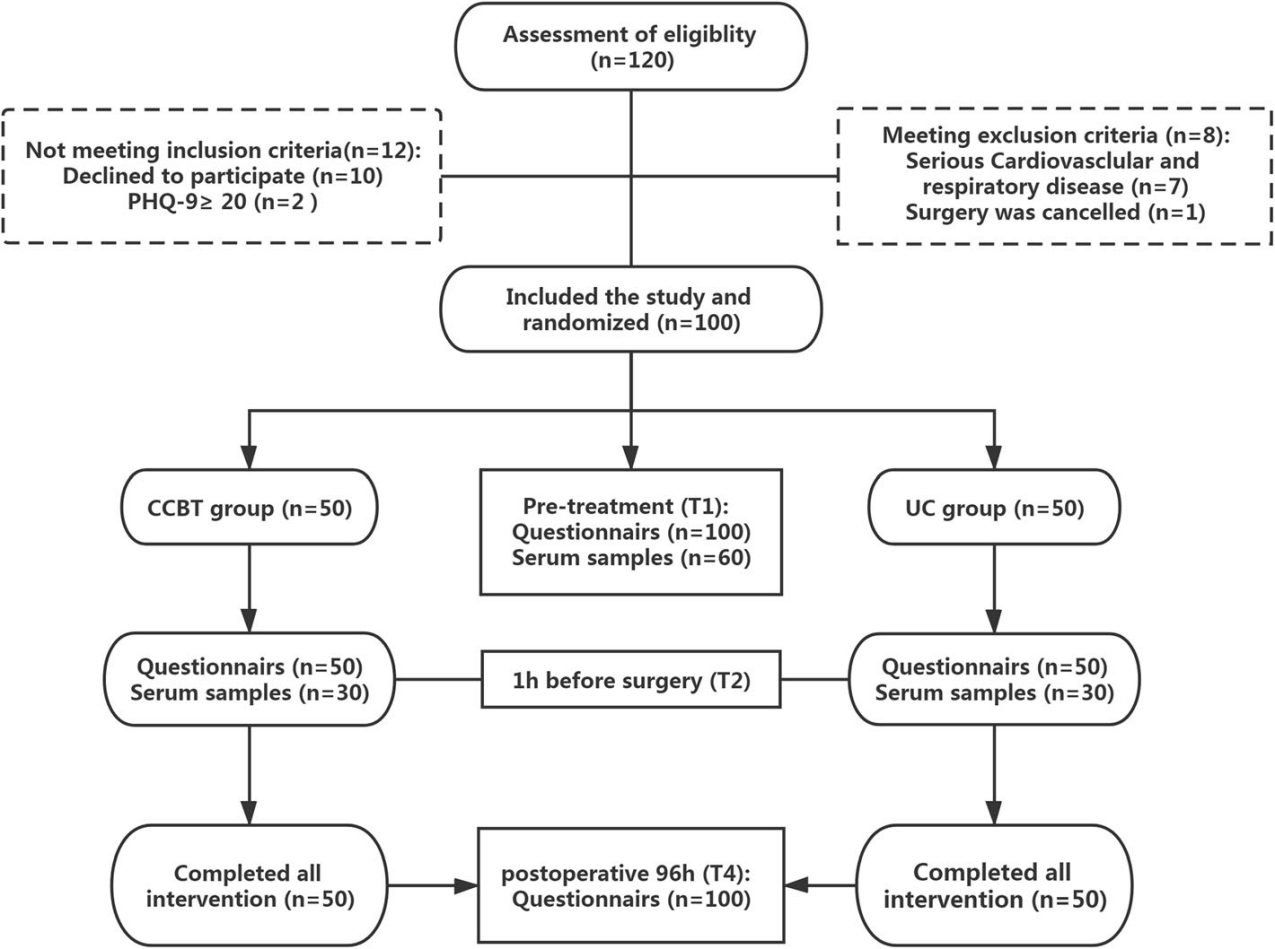
CONSORT flow diagram

One hundred twenty patients were interested in participation and assessed eligibility. Following inclusion and exclusion criteria, finally 100 patients were approached for the study. Figure 1 shows the CONSORT flow diagram, including information about study exclusion and the sample size for analysis. Table 1 summarizes the demographic data that participants were well-matched in terms of baseline characteristics between the two groups.

**Table 1:** Characteristics of the study population

Variable	UC group (*n* = 50)	CCBT group (*n* = 50)	*p*
Age (mean ± SD)	43.08 ± 12.51	42.10 ± 12.89	0.70
Gender (%)			
Men	25 (50.00)	22 (44.00)	0.55
Women	25 (50.00)	28 (56.00)	
Education (%)			
High school graduate	36 (72.00)	37 (74.00)	0.82
Non-graduates	14 (28.00)	13 (26.00)	
TAI (mean ± SD)	34.50 ± 8.01	35.18 ± 9.51	0.87
Diagnosis (%)			
CRS with NP	26(52.00)	30(60.00)	0.574
CRS (only)	20(40.00)	15(30.00)	
Other	4(8.00)	5(10.00)	

All values are mean ± SD or number (proportion). *TAI* Spielberger Trait Anxiety Inventory. *CRS with NP* chronic rhinosinusitis with nasal polyps, *CRS* chronic rhinosinusitis

### Comparative analysis between two groups on the SAI, PHQ-9, and AIS scores

The results of repeated-measures ANOVA found group*time interaction and time effect for SAI and AIS scores was statistically significant (all *p* < 0.01). Further simple effect analysis found, to compare with UC group, the mean scores of SAI in CCBT group were lower and had a difference value 5.04 (95% CI − 7.50, − 2.54) and 5.64 (95% CI − 8.01, − 3.02) at T2 and T3 (all *p* < 0.01). The mean scores of AIS in CCBT group were lower and had a difference value 1.26 (95% CI − 2.05, − 0.47) and 1.26 (95% CI − 2.09, − 0.50) at T3 and T4 (all *p* < 0.01). For PHQ-9 scores, group*time interaction was not statistically significant (*p* = 0.06) and time effect was statistically significant (*p* < 0.01) (see Table 2).

**Table 2:** Between-group differences on study outcome measures

Measure	UC group (*n* = 50)	CCBT group (*n* = 50)	Difference (95% CI)	*p*^*d*^
**SAI**				
T1	32.02 ± 5.89	33.12 ± 6.80	1.10 (− 1.35, 3.61)	0.39
T2	44.80 ± 7.06^e^	39.76 ± 4.95^e^	− 5.04 (− 7.04, − 2.63)	< 0.01
T4	28.98 ± 5.47^e,f^	23.34 ± 3.37^e,f^	− 5.64 (− 7.44, − 3.84)	< 0.01
*p*	< 0.01^a^	< 0.01^b^	< 0.01^c^	
**PHQ-9**				
T1	3.40 ± 3.26	3.14 ± 2.48	− 0.26 (− 1.32, 0.92)	0.66
T3	3.98 ± 2.91	4.18 ± 2.50	− 0.20 (− 1.26, 0.98)	0.71
T4	2.08 ± 1.90	1.98 ± 1.73	0.10 (− 0.94, 1.31)	0.78
*p*	0.81^a^	< 0.01 ^b^	0.06^c^	
**AIS**				
T1	3.16 ± 2.44	3.60 ± 2.16	0.44 (− 0.47, 1.34)	0.91
T3	6.16 ± 2.21^e^	4.90 ± 1.73^e^	− 1.26 (− 2.05, − 0.47)	0.002
T4	3.18 ± 2.22^g^	1.92 ± 1.86^e,g^	− 1.26 (− 2.07, − 0.45)	0.003
*p*	0.005^a^	< 0.01 ^b^	< 0.01^c^	

*SAI* State Anxiety Inventory, *PHQ-9* Patient Health Questionnaire Depression Scale-9 item, *AIS* Athens Insomnia Scale, *T1* before the intervention, *T2* at 1 h before surgery, *T3* at postoperative 48 h, *T4* at postoperative 96 h (after the intervention completed)

^a^*p* value of group effect

^b^*p* value of time effect

^c^*p* value of interactive effects between time and group

^d^*p* value adjusted by Bonferroni method

^e^Compared with the T1, *p* < 0.05, adjusted by Bonferroni method

^f^Compared with the T2, *p* < 0.05, adjusted by Bonferroni method

^g^Compared with the T3, *p* < 0.05, adjusted by Bonferroni method

### The comparison about the positive rate of participants

**Fig. 2:**
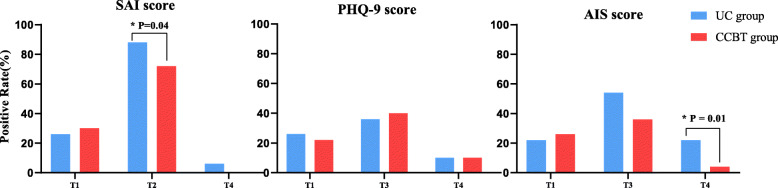
The comparison about the positive rate of participants

The results of the comparison about positive rate of participants who were moderate and severe anxiety (SAI score > 37) illustrated that the difference was statistically significant between the two group (72% vs. 88%, *p* = 0.04). The results of the comparison about positive rate of participants who were insomnia (AIS score > 5) illustrated that the difference after intervention was statistically significant between the two groups (4% vs 22%, *p* = 0.01), while the results show no difference about the positive rate for depression (PHQ-9 > 4) between two groups (Fig. 2).

### The comparison about duration of surgery, anesthesia, and PACU between-two groups

The duration of surgery between-two groups had no difference significantly (*p* = 0.583). The duration of anesthesia and PACU in the CCBT group were shorter than that of the UC group (*p* = 0.001 and *p* < 0.001) (see Table 3).

**Table 3:** The duration comparative analysis between two groups(mean ± SD)

Duration (min)	UC group (*n* = 50)	CCBT group (*n* = 50)	*p*
Surgery	137.78 ± 18.41	139.80 ± 18.25	0.58
Anesthesia	214.68 ± 20.55	199.24 ± 22.79	0.001
PACU	32.20 ± 8.84	22.98 ± 8.26	< 0.01

*PACU* post-anesthesia care unit, *Duration of anesthesia* time between the induction and leaving from PACU, *Duration of surgery* time between touching the skin and the end of suture or tamponade, *Duration of PACU* time between PACU admission to discharge

### The comparison of Cortisol and ACTH levels between-two groups

The cortisol level of the UC group were significantly greater at T2 compared to T1 (*p* = 0.001). At T2, the cortisol level were higher significantly in the UC group than that of the CCBT group (*p* = 0.02) (see Table 4).

**Table 4:** Cortisol and ACTH comparative analysis between two groups(mean ± SD)

Measure	UC (*n* = 30)		CCBT (*n* = 30)	
	T1	T2	T1	T2
Cortisol (nmol/L)	295.43 ± 125.66	349.57 ± 83.39∆*	307.07 ± 111.92	306.22 ± 74.86
ACTH (pg/mL)	38.17 ± 17.40	40.57 ± 13.41	37.39 ± 24.70	39.55 ± 20.30

*T1* before the intervention, *T2* at 1 h before surgery

*p < 0.05, *p* value of independent-samples t test

∆*p* < 0.05, *p* value of paired t test

### The change trend comparison about SBP, DBP, and HR

**Fig. 3:**
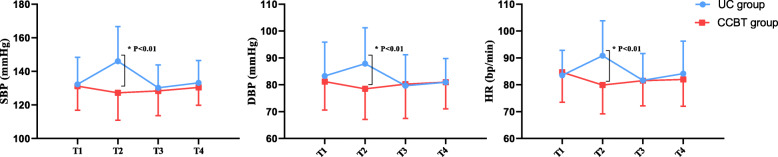
The change trend line chart of BP and HR

The change trend comparison about SBP, DBP, and HR between two groups found that the trend for CCBT group was more stabilized than the UC group. More importantly, at 1 h before surgery, the SBP mean value in CCBT group was lower than that of the UC group (127.24 ± 16.27 vs 146.06 ± 20.59, *p* < 0.01), the same with the DBP mean value (78.48 ± 11.42 vs 87.86 ± 13.31, *p* < 0.01) and the HR mean value (79.90 ± 10.73 vs 90.42 ± 12.83, *p* < 0.01) (Fig. 3).

### Satisfaction survey comparisons between two groups

Participant satisfaction was significantly higher in the CCBT group than in the UC group for all of the assessed categories except disease care (*p* = 0.12) before discharge care item (Table 5).

**Table 5:** Satisfaction comparison between two groups before discharge, median (P_25_, P_75_)

Item	UC group (*n* = 50)	CCBT group (*n* = 50)	*p*
Psychological care	8(7, 10)	10(9, 10)	< 0.01
Disease care	20(18, 20)	20(18, 20)	0.12
Psychosomatic management	15(14, 17.5)	20(16.5, 20)	< 0.01
Total	43(40, 45.5)	48(45, 50)	< 0.01

## Discussion

In the present study, we aimed to explore the efficacy of an optimized five-sessions cCBT for individuals with FESS who had psychobiological responses because of surgery. The intervention appeared to be useful not only in improving anxiety and insomnia, but also in biological parameters (cortisol, hemodynamic control) compared to the control group. However, there were few RCTs reported about cCBT treating FESS patients with psychological distress. The present study provides preliminary data to assess the potential efficacy and mechanism of mobile technology-based cCBT interventions in clinical surgery applications.

In the present study, the anxiety level increased before surgery in both groups. The causes may be the lack of an adequate preoperative education, awareness of the process of surgery and anesthesia, and uncertainties about postoperative steps and worrying about the outcomes. The newly cCBT based on nursing education can help patients to know surgery and anesthesia adequately, and preview postoperative rehabilitation in advance. The result showed the CCBT group experienced a lower level of anxiety and insomnia than the control group. The improvement in insomnia may be affected by the improvement of anxiety. The results were consistent with the results of recent meta-analyses, and cCBT was found to improve self-reported psychological outcomes (anxiety and depression) and physical outcomes (pain and insomnia) of patients with psychiatric and somatic disorders (Mehta et al. 2019). However, no significant effects were observed for depression symptoms between the two groups. It may be due to surgery-related stress being experienced on a short-term timescale, while depression may exhibit a more extended time course (Yang et al., 2015). The mechanisms that psychological interventions can influence anxiety in surgery patients may be affected underlying the neuroendocrine response to surgical stress (Villa et al. 2020).

The surgery-related perioperative stress may affect extensively patients’ neuroendocrine pathways (Maduka et al. 2015). Enhanced cognitive coping may modulate hypothalamic-pituitary-adrenal (HPA) axis activity and improve the levels of endocrine hormones (Abelson et al. 2008). Anxiety can increase the cortisol secretion, which has been observed among patients awaiting operation. The newly developed cCBT based on CBT and nursing education can significantly reduce cortisol levels of the CCBT group, which may be due to cognitive bias modification and behavioral coping in CBT. Cortisol homeostasis is essential for cognitive and affective functions (O'Connor et al. 2021). Increased cortisol and an imbalance of the HPA axis can contribute to the development of anxiety and depression (van Dalfsen and Markus, 2018). The differences of ACTH were not observed and it may illustrate that ACTH is not a sensitive index related-surgery stress than cortisol. The connection among emotions, hormones, and brain networks is the basis for subsequent research.

Numerous studies and meta-analyses have linked BP responses to mental stress to poor health outcomes (O'Connor et al. 2021). Generally, anxiety increases blood pressure, systemic vascular resistance, sympathetic activity (Pan et al. 2015). Preoperative stable hemodynamics contributes to control hypotension and reduce intraoperative bleeding which help to complete the surgery smoothly and lighten the burden on the anesthesiologist. Gu et al. in their study relieved anxiety and stabilized BP and HR by dexmedetomidine combined with parecoxib before anesthesia induction in patients for FESS (Gu et al., 2020). In this study, the patients in the CCBT group had a lower preoperative BP and HR, which showed cCBT can help anesthesiologists control high BP and HR using minimal drugs. The shorter duration of anesthesia and PACU illustrated patients using cCBT program experienced shorter duration of anesthesia induction and faster postoperative recovery time. These are key factors of improved perioperative outcomes and can also ease the burden for anesthetists (Sgrò et al. 2019), while the duration of surgery was no difference between two groups, which may be related to different surgeons. In the present study, cCBT had a positive effect on stabilizing hemodynamics before anesthesia induction, but had a limited impact on vital signs during the postoperative recovery period.

Making the patient safe and comfortable during the whole perioperative period is our medical aim. Satisfaction scores were universally high in the CCBT group. However, differences in Disease Care item was not observed, which may be correlated to accept standard nursing procedures. The patients participating in the CCBT group indicated that they received more services, especially psychological services. The more satisfactory medical experience makes the relationship between medical staff and patients harmonious and reduces the incidence of violence (Meng et al. 2020).

The study had several limitations. Firstly, the intervention was conducted at a single site, with a relatively small sample size, and only a part of participants were willing to be blood collected, which may have been insufficient and subject to type II errors during analysis. Secondly, our study did not assess the long-term effects in patients, which can be rectified in a future study that continues to follow up on these patients and collect additional data. Thirdly, we did not measure intraop blood loss or surgeon’s satisfaction of a bloodless field, which are important measures to evaluate the success of FESS. Two teams of surgeons may also introduce heterogeneity because of different experience. Finally, further research direction will classify different populations and optimize the cCBT program to become more personalized and targeted.

## Conclusion

In conclusion, we found that the optimized cCBT program may be a suitable and effective adjunctive treatment relieving psychobiological responses for patients undergoing FESS. With the help of multidisciplinary treatment, the new form of psychosomatic intervention can improve the access to psychological services and surgical outcomes.

## Supplementary Information

**Additional file 1:Supplementary Table 1.** State Trait Anxiety Scale (STAI). **Supplementary Table 2.** Patients Health Questionnaire-9 (PHQ-9). **Supplementary Table 3.** Athens Insomnia Scale (AIS). **Supplementary Table 4.** Satisfaction Survey Questionnaire.

## Data Availability

The dataset used and analyzed during the current study is available from the corresponding author on reasonable request.

## Abbreviations

FESS: Functional endoscopic sinus surgery
CRS: Chronic rhino sinusitis
TIVA: Total intravenous anesthesia
CBT: Cognitive behavior therapy
CCBT: Computer-assisted cognitive-behavior therapy
UC: Usual-care
ERAS: Enhanced recovery after surgery
SAI: State anxiety inventory
TAI: Trait anxiety inventory
PHQ-9: Patients health questionnaire depression scale-9 item
AIS: Athens insomnia scale
ACTH: Adrenocorticotropic hormone
HPA: Hypothalamic-pituitary-adrenal
CLIA: Chemiluminescence assay
SBP: Systolic blood pressure
DBP: Diastolic blood pressure
HR: Heart rate
PACU: Post-anesthesia care unit
