# Supplementary material for: The efficacy of computer-assisted cognitive behavioral therapy (cCBT) on psychobiological responses and perioperative outcomes in patients undergoing functional endoscopic sinus surgery: a randomized controlled trial
Source: Perioper Med (Lond). 2021 Aug 19;10:28. doi: 10.1186/s13741-021-00195-3 (PMC8375045; doi:10.1186/s13741-021-00195-3)
Supplement: Supplementary file 1 — Additional file 1:Supplementary Table 1. State Trait Anxiety Scale (STAI). Supplementary Table 2. Patients Health Questionnaire-9 (PHQ-9). Supplementary Table 3. Athens Insomnia Scale (AIS). Supplementary Table 4. Satisfaction Survey Questionnaire. [file 13741_2021_195_MOESM1_ESM.docx]

**Supplementary table 1** State Trait Anxiety Scale (STAI)

**State Trait Anxiety Scale (STAI)**

Instructions: Please read each statement carefully, and then choose the answer you think is most appropriate below, indicating all the feelings you have after learning about the upcoming surgery. There is no right or wrong way, and don't spend too much time thinking about any one statement.

| N | Content | Answer | | | |
| --- | --- | --- | --- | --- | --- |
|  |  | Not at all | Some what | Moderately so | Very much so |
| 1* | I feel at peace | 4 | 3 | 2 | 1 |
| 2* | I feel safe | 4 | 3 | 2 | 1 |
| 3 | I feel nervous | 1 | 2 | 3 | 4 |
| 4 | I feel limited | 1 | 2 | 3 | 4 |
| 5* | I feel at easy | 4 | 3 | 2 | 1 |
| 6 | I feel upset | 1 | 2 | 3 | 4 |
| 7 | I am fretting over the possibility of misfortune | 1 | 2 | 3 | 4 |
| 8* | - I feel satisfied | 4 | 3 | 2 | 1 |
| 9 | I feel scared | 1 | 2 | 3 | 4 |
| 10* | I feel comfortable | 4 | 3 | 2 | 1 |
| 11* | I feel confident | 4 | 3 | 2 | 1 |
| 12 | I feel jittery | 1 | 2 | 3 | 4 |
| 13 | I was on tenterhooks | 1 | 2 | 3 | 4 |
| 14 | - I feel indecision | 1 | 2 | 3 | 4 |
| 15* | I feel at ease | 4 | 3 | 2 | 1 |
| 16* | I feel perfectly satisfied | 4 | 3 | 2 | 1 |
| 17 | I am in trouble | 1 | 2 | 3 | 4 |
| 18 | I feel stressed out | 1 | 2 | 3 | 4 |
| 19* | I feel calm | 4 | 3 | 2 | 1 |
| 20* | I feel happy | 4 | 3 | 2 | 1 |
| SAI score： | | | | | |
|  |  | Almost never | Sometimes | Often | Almost always |
| 21* | I always feel happy | 4 | 3 | 2 | 1 |
| 22 | I always feel nervous | 1 | 2 | 3 | 4 |
| 23* | I feel self-satisfied | 4 | 3 | 2 | 1 |
| 24* | I wish I could be as happy as everyone else | 4 | 3 | 2 | 1 |
| 25 | I feel like I'm collapsing | 1 | 2 | 3 | 4 |
| 26* | I feel very calm | 4 | 3 | 2 | 1 |
| 27* | I am peaceful, calm | 4 | 3 | 2 | 1 |
| 28 | I find the difficulties mounting, too many to overcome | 1 | 2 | 3 | 4 |
| 29 | I worry too much about things that don't matter | 1 | 2 | 3 | 4 |
| 30* | I am happy | 4 | 3 | 2 | 1 |
| 31 | My mind is in a state of confusion | 1 | 2 | 3 | 4 |
| 32 | I lack confidence | 1 | 2 | 3 | 4 |
| 33* | I feel safe | 4 | 3 | 2 | 1 |
| 34* | I'm easy to decide | 4 | 3 | 2 | 1 |
| 35 | I don't feel right | 1 | 2 | 3 | 4 |
| 36* | I feel very satisfied | 4 | 3 | 2 | 1 |
| 37 | Some insignificant thoughts always haunt me and disturb me | 1 | 2 | 3 | 4 |
| 38 | I tend to have depressed thoughts and I can't get rid of them easily | 1 | 2 | 3 | 4 |
| 39* | I am a calm person | 4 | 3 | 2 | 1 |
| 40 | When I think about my current state, I get nervous easily | 1 | 2 | 3 | 4 |
| TAI score： | | | | | |

**Supplementary table 2** Patients Health Questionnaire-9 (PHQ-9)

**Patients Health Questionnaire-9 (PHQ-9)**

Answer according to the following 9 questions, with √ above the number that matches your choice:

| N | How often have the following bothered you in the past two weeks? | Score | | | |
| --- | --- | --- | --- | --- | --- |
|  |  | Not at all | Some days | More than half the days数 | Almost everyday |
| 1 | Lacking enthusiasm or interest in doing things | 0 | 1 | 2 | 3 |
| 2 | To feel low, depressed, or hopeless | 0 | 1 | 2 | 3 |
| 3 | Difficulty falling asleep, restlessness, or excessive sleep | 0 | 1 | 2 | 3 |
| 4 | Feeling tired or lacking in energy | 0 | 1 | 2 | 3 |
| 5 | Loss of appetite or eating too much | 0 | 1 | 2 | 3 |
| 6 | Feeling bad or a failure, or letting yourself or your family down | 0 | 1 | 2 | 3 |
| 7 | You have trouble focusing on things, such as reading a newspaper or watching television | 0 | 1 | 2 | 3 |
| 8 | Slow enough in movement or speech to be noticed? Or just the opposite - fidgeting or fidgeting and fidgeting more than usual | 0 | 1 | 2 | 3 |
| 9 | It's better to die or hurt yourself in some way | 0 | 1 | 2 | 3 |
| The highest score is 27 points, the lowest score is 0 points, and 5 points or more is meaningful 得分： | | | | | |

**Supplementary table 3** Athens Insomnia Scale (AIS)

**Athens Insomnia Scale (AIS)**

Instructions: This scale evaluates your sleep condition through your subjective feelings about sleep. If the problems listed below have happened to you at least 3 times in the past week, please select the appropriate self-assessment result.

| N | Content | Score | | | |
| --- | --- | --- | --- | --- | --- |
|  |  | 0 | 1 | 2 | 3 |
| 1 | the time to fall asleep (Between the time you turn off the lights and fall asleep) | No problem | A slight delay | Significant delay | Severe delay or no sleep |
| 2 | Waking up at night | No problem | A slight delay | Significant delay | Severe delay or no sleep |
| 3 | Wake up earlier than you want | No problem | A slight delay | Significant delay | Severe delay or no sleep |
| 4 | total sleep time | enough | Slightly less than | inadequate | Severe insufficient or no sleep |
| 5 | Total sleep quality (no matter how long) | satisfaction | Mild dissatisfaction | Significant dissatisfaction | Serious dissatisfaction or no sleep |
| 6 | Emotions during the day | normal | Mild depression | Significantly depressed | Severe depression |
| 7 | Daytime physical function (physical or mental: memory, cognition, attention, etc.) | enough | marginal effect | obvious significance | touch severely |
| 8 | daytime sleepiness | Not at all | Slight drowsiness | Significant drowsiness | Serious drowsiness |

Score criteria: total score less than 4 points: no sleep disorder; If the total score is 4-6: Suspicious insomnia; If the total score is above 6: Insomnia. According to the total score, those with > score of 6 were considered positive.

**Supplementary table 4** Satisfaction Survey Questionnaire

**Satisfaction Survey Questionnaire**

| ID | Content | Very goog | good | general | bad | Very bad |
| --- | --- | --- | --- | --- | --- | --- |
|  |  | 5 | 4 | 3 | 2 | 1 |
|  | **Satisfaction of psychological care services** |  |  |  |  |  |
| 1 | psychological assessment |  |  |  |  |  |
| 2 | Effect of psychological care |  |  |  |  |  |
|  | **Satisfaction of disease care services** |  |  |  |  |  |
| 3 | Health education during perioperative period |  |  |  |  |  |
| 4 | Satisfactory responses about disease |  |  |  |  |  |
| 5 | Postoperative disease care |  |  |  |  |  |
| 6 | Effect of disease care |  |  |  |  |  |
|  | **Psychosomatic symptom management satisfaction** |  |  |  |  |  |
| 7 | Understanding of the operation during the perioperative period |  |  |  |  |  |
| 8 | Emotional state management during perioperative period |  |  |  |  |  |
| 9 | Pain management during perioperative period |  |  |  |  |  |
| 10 | Sleep management during perioperative period |  |  |  |  |  |
| Suggestion： | | | | | |  |

All items are used 5-point Likert scale strategy. (0 = very bad, 1= bad, 2 = general, 3 = good, 4 = very good)
